# Psychiatric morbidity and its impact on surgical outcomes for esophageal and gastric cancer patients: A nationwide cohort study

**DOI:** 10.18632/oncotarget.18347

**Published:** 2017-06-02

**Authors:** Huan Song, Jianwei Zhu, Donghao Lu, Fang Fang, Weimin Ye, Lars Lundell, Jan Johansson, Mats Lindblad, Magnus Nilsson

**Affiliations:** ^1^ Department of Medical Epidemiology and Biostatistics, Karolinska Institutet, Stockholm, Sweden; ^2^ Division of Surgery, CLINTEC, Department of Surgical Gastroenterology, Karolinska Institute at Karolinska University Hospital, Stockholm, Sweden; ^3^ Department of Surgery, Skåne University Hospital, Lund, Sweden

**Keywords:** psychiatric morbidity, prognosis, surgery, gastric cancer, esophageal cancer

## Abstract

**Background:**

Due to the lack of detailed clinical information, existed evidence regarding a link between psychiatric factors and adverse cancer prognosis was inclusive.

**Results:**

We identified 1,340 patients (48.8%) with perioperative psychiatric morbidity. Preoperative psychiatric morbidity was significantly associated with both general and surgical complications within 30 days (RR = 1.3, 95% confidence interval [CI] 1.1–1.5), and the risk of death within 90 days (RR = 1.6; 95% CI 1.1–2.2) after surgery. The hazards for mortality beyond 90 days was approximately 2-fold increased among patients with perioperative psychiatric morbidity (HR = 2.0, 95% CI 1.7–2.3 for overall mortality).

**Materials and Methods:**

Based on the Swedish National Registry for Esophageal and Gastric cancer (NREV), we constructed a nationwide prospective cohort containing 2,745 surgically treated patients in 2006–2012. Perioperative psychiatric morbidity was defined as a clinical diagnosis of psychiatric disorder, from two years before to two years after surgery. Using propensity scores, we applied inverse probability of treatment weights (IPTW)-weighted Poisson regression model to evaluate relative risk (RR) of short-term surgical outcomes in relation to perioperative psychiatric morbidity. Further, IPTW-weighted Cox proportional hazards model was used to estimate hazard ratios (HRs) for mortality that occurred after 90 days of surgery.

**Conclusions:**

Perioperative psychiatric morbidity could worsen both short-term and long-term surgical outcomes among patients with gastric or esophageal cancer.

## INTRODUCTION

An increased risk of experiencing multiple psychiatric disorders has been reported among patients with cancer, from the pre-diagnostic period to the period after therapy initiation [1, 2]. The risk elevation, albeit varied in magnitude across studies, consistently pertained in a variety of cancers, different countries, and among both young and old patients [2–9]. Consequently, during recent years, growing attention has been paid to the question whether or not the co-occurrence of these psychiatric events could affect the outcome of cancer treatment [10].

There is accumulating evidence, from both observational [11] studies and animal models [12], indicating a link between psychiatric factors and adverse cancer prognosis, without taking the treatment modalities into consideration. Recently, a Swedish study [13] showed that the co-occurring psychiatric disorders (psychiatric morbidity) were related to poorer survival in surgically treated esophageal cancer patients. These observations, however, were based on data retrieved from public registries, where information on personal/tumor characteristics is limited. Given that the outcomes of surgery can be influenced by many clinical factors and treatment details, it is therefore of profound interest to re-examine the association between psychiatric morbidity and survival outcomes among operated cancer patients, using prospectively collected data which captured detailed information on patient characteristics, cancer treatments, and outcomes. In addition, the impact of psychiatric morbidity on short-term surgical outcomes, such as severe complications, has rarely been assessed. To the end, we leveraged a nationwide prospective cohort of esophageal and gastric cancer patients with rich clinical information to elucidate the association of psychiatric morbidity with both short-term and long-term surgical outcomes.

## RESULTS

Table 1 illustrates the basic characteristics of the 2,745 eligible participants, together with their disease- or treatment- related details. The average age at cancer diagnosis was 68.3 years; and there were more males (65%), and those with a gastric cancer diagnosis (60%). Majority of the patients had stage I–III cancer disease (90%) and were in physical status ASA I–II (74%), which corresponded well to the fact that 81% of the patients had operations with curative intention.

**Table 1:** Characteristics of all participants and the subgroup of patients with or without psychiatric comorbidity

Characteristics	Overall (*n* = 2,745)	Any psychiatric comorbidity*	
		No (*n* = 1,405)	Yes (*n* = 1,340)
**Demographic factors**			
Age, mean ± SD, years	68.3 ± 11.1	69.1 ± 10.9	67.4 ± 11.2
Gender (% male)	65.7	68.7	62.5
Marital status, *n* (%)			
Single	315 (11.5)	155 (11.0)	160 (11.9)
Married	1579 (57.5)	825 (58.7)	754 (56.3)
Divorce	429 (15.6)	202 (14.4)	227 (16.9)
Widow/widower	422 (15.4)	223 (15.9)	199 (14.9)
Education level,*n* (%)			
≤ 9 years	1072 (39.1)	552 (39.3)	520 (38.8)
10–12 years	1085 (39.5)	555 (39.5)	530 (39.6)
≥ 12 years	483 (17.6)	249 (17.7)	234 (17.5)
Missing	105 (3.8)	49 (3.5)	56 (4.2)
History of psychiatric disorder^†^, *n* (%)			
No	2184 (80.0)	1284 (91.4)	900 (67.2)
Yes	561 (20.0)	121 (8.6)	440 (32.8)
**Disease-related factors**			
Time of follow-up (from operation to death, emigration or the end of study)			
Mean ± SD, months	26.1 ± 22.3	28.1 ± 23.1	24.2 ± 21.2
ASA physical status, *n* (%)			
I–II	2033 (74.1)	1062 (75.6)	971 (72.5)
III–IV	571 (20.8)	279 (19.9)	292 (21.8)
Missing	141 (5.14)	64 (4.5)	77 (5.7)
Cancer type, *n*(%)			
Gastric cancer	1637 (59.6)	877 (62.4)	760 (56.7)
Esophageal cancer	1108 (40.4)	528 (37.6)	580 (43.3)
Cancer stage, *n*(%)			
Stage 0	93 (3.39)	50 (3.56)	43 (3.21)
Stage I	739 (26.9)	388 (27.6)	351 (26.2)
Stage II	569 (20.7)	288 (20.5)	281 (21.0)
Stage III	1167 (42.5)	592 (42.1)	575 (42.9)
Stage IV	100 (3.64)	49 (3.49)	51 (3.81)
Missing	77 (2.81)	38 (2.7)	39 (2.9)
Charlson comorbidity index, *n* (%)			
0–2	1619 (59.0)	818 (58.2)	801 (59.8)
≥ 3	1126 (41.0)	587 (41.8)	539 (40.2)
**Treatment details**			
Hospital Volume, *n* (%)			
Low (< 20 cases/year)	319 (11.6)	171 (12.2)	148 (11.0)
Median (20–40 cases/year)	321 (11.7)	157 (11.2)	164 (12.2)
High (> 40 cases/year)	2104 (76.7)	1077 (76.6)	1027 (76.7)
Missing	1 (0.1)	0 (0.0)	1 (0.1)
Preoperative chemotherapy, *n* (%)			
No	1722 (62.7)	922 (65.6)	800 (59.7)
Yes	794 (28.9)	376 (26.8)	418 (31.2)
Missing	229 (8.34)	107 (7.6)	122 (9.1)
Preoperative radiotherapy, *n*(%)			
No	2227 (81.1)	1171 (83.4)	1056 (78.8)
Yes	284 (10.4)	125 (8.90)	159 (11.9)
Missing	234 (8.52)	109 (7.8)	125 (9.3)
Operation type,*n* (%)			
Curative	2222 (81.0)	1157 (82.4)	1065 (79.5)
Palliative	284 (10.4)	141 (10.0)	143 (10.7)
Missing	239 (8.71)	107 (7.62)	132 (9.85)

^*^ Perioperative psychiatric morbidity was defined as a clinical diagnosis of psychiatric disorder through hospital visit or prescription of psychiatric medications, from two years before and to two years after surgery.

^†^History of psychiatric disorders was determined by psychiatric diagnosis or use of psychiatric medications detected more than two years before operation.

We identified in total 1,340 individuals (48.8% of all operated patients) with the defined perioperative psychiatric morbidity, including 230 patients with preoperative alone (17.2%), 560 with pre-and post-operative (continued; 41.8%), and 550 with postoperative alone (41.0%) psychiatric morbidity. Esophageal cancer patients had higher possibility of psychiatric morbidity than gastric cancer patients (Table 1). Patients with psychiatric morbidity had also higher possibility of a positive history of psychiatric morbidity more than two years before operation (32.8%), compared to patients without perioperative psychiatric morbidity (8.6%).

**Figure 1:**
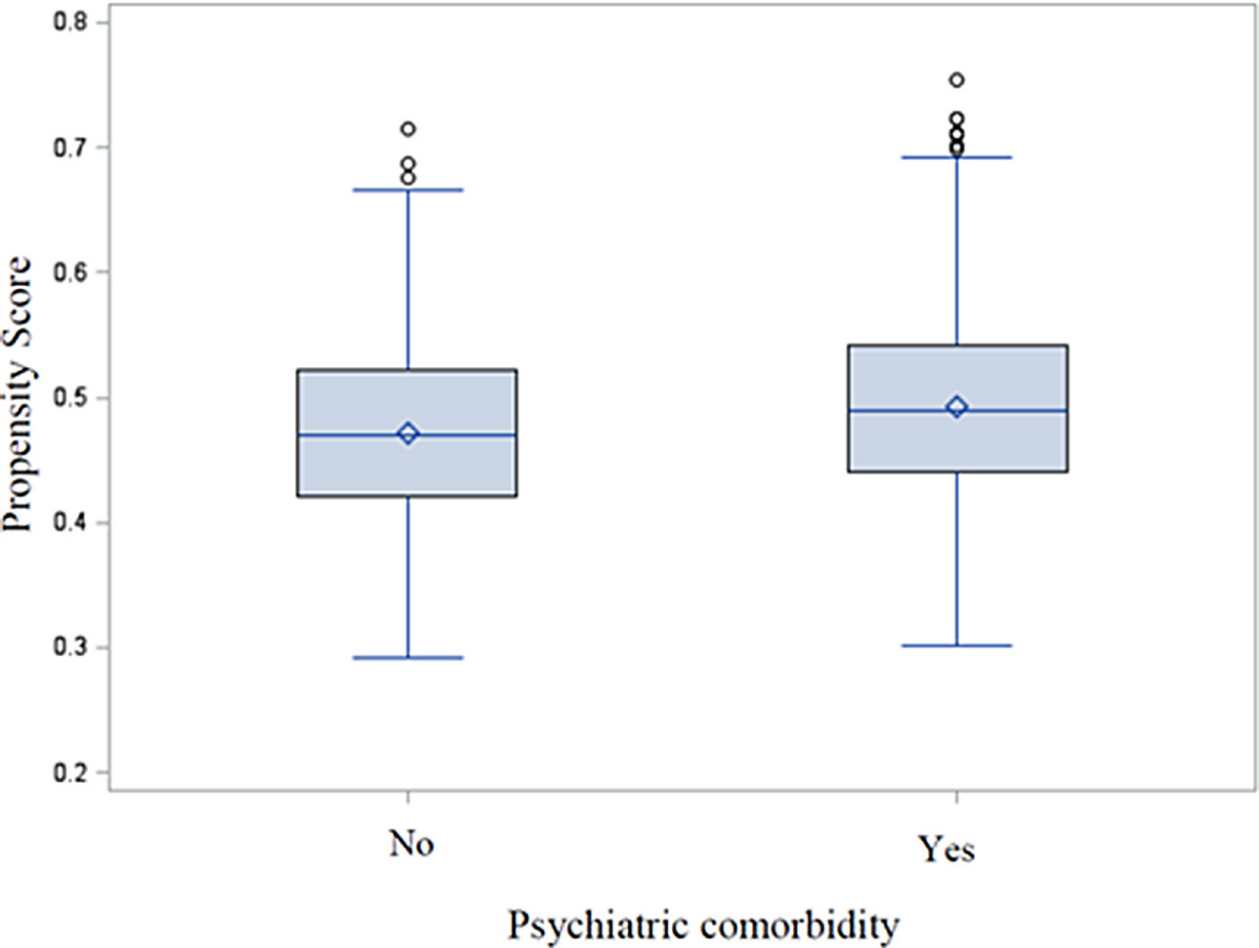
Evaluation of common support using distribution of propensity scores for exposed and unexposed groups

Using logistic regression, we modeled the probability of suffering any perioperative psychiatric morbidity to generate propensity scores. Although the nature of our observational data resulted in a difference, the large overlapping range in propensity score distribution between exposure groups (shown in Figure 1) displayed a general support for the propensity model as such. Moreover, following the application of IPTW, balanced covariate between groups was demonstrated by the low standardized bias (< 0.25) for each involved covariate in weighted data.

Table 2 shows the proportions of short-term surgical outcomes, along with the adjusted RRs describing their associations with preoperative psychiatric morbidity. Overall, preoperative psychiatric morbidity was associated with both general (RR = 1.28, 95% CI 1.03–1.60) and surgical (RR = 1.29, 95% CI 1.05–1.60) complications within 30 days. More specifically, except for abscess, all other outcomes had 17%–62% excess risks in relation to preoperative psychiatric morbidity, although many were not statistically significant due to the small numbers of outcomes. No association of preoperative psychiatric morbidity was noted for the length of hospital stay. An increased risk of short-term mortality after surgery, especially 90-day mortality, was noted among patients with preoperative psychiatric morbidity (RR = 1.57, 95% CI 1.14–2.17 for 90-day mortality). The association, however, largely attenuated to almost null (RR = 1.03, 95% CI 0.69–1.52) after further adjusting for general and surgical complications within 30 days after surgery.

**Table 2:** Associations between preoperative psychiatric morbidity and short-term surgery outcomes (n = 2,745)

Short-term outcomes	Preoperative psychiatric morbidity		
	Yes	No	Relative risk (RR)*
**General complication within 30 days (missing = 234)**	170/715 (23.8%)	330/1797 (18.4%)	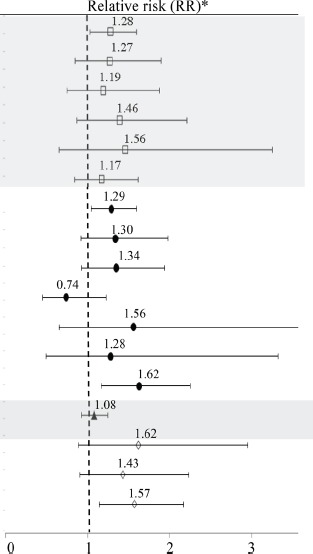
Severe pneumonia	52/715 (7.4%)	95/1797 (5.3%)	
Sepsis	36/715 (5.0%)	72/1797 (4.0%)	
Severe cardiovascular complications	34/715 (4.8%)	66/1797 (3.7%)	
Pulmonary embolism	13/715 (1.8%)	21/1797 (1.2%)	
Other severe general complications	73/715 (10.2%)	147/1797 (8.2%)	
**Surgical complication within 30 days (missing = 231)**	184/718 (25.6%)	361/1797 (20.1%)	
Hemorrhage	18/718 (2.5%)	55/1797 (3.1%)	
Anastomotic leak	54/718 (7.5%)	102/1797 (5.7%)	
Abscess	34/718 (4.7%)	84/1797 (4.7%)	
Thoracic duct injury	14/718 (2.0%)	17/1797 (0.9%)	
Nerve palsy	10/718 (1.4%)	22/1797 (1.2%)	
Other severe surgical complications	88/718 (12.3%)	131/1797 (7.3%)	
**Hospital stay longer than 2 weeks (missing = 230)**	385/720 (53.5%)	846/1796 (47.1%)	
Death within 30 days	28/790 (3.5%)	42/1956 (2.1%)	
Death within 60 days	48/790 (6.1%)	78/1956 (4.0%)	
**Death within 90 days**	72/790 (9.1%)	116/1956 (5.9%)	

^*^ Relative risk was estimation by inverse probability of treatment weights (IPTW) weighted Poisson regression model, weighed by propensity score and adjusted for hospital volume (low, median, or high), preoperative chemotherapy (yes/no), preoperative radiotherapy (yes/no), operation type (curative/palliative), history of mental disorder (yes/no).

Similar findings were noted for preoperative psychiatric morbidity identified through hospital visit or medication, with the exception of the null association of hospital diagnosis with general postoperative complications within 30 days (Table 3). Subgroup analyses by previous history of psychiatric disorders obtained similar results as the main analyses.

**Table 3:** Associations between different types of preoperative psychiatric morbidity and short-term surgery outcomes

Preoperative psychiatric morbidity	General complication within 30 days (missing = 234)		Surgical complication within 30 days (missing = 231)		Death within 90 days	
	Number of event/total (%)	RR bold>(95% CI)*	Number of event/ total (%)	RR (95% CI)*	Number of event/ total (%)	RR (95% CI)*
**Specified by identification approach**						
No	330/1797 (18.4%)	Reference	361/1797(20.1%)	Reference	116/1956(5.9%)	Reference
Psychiatric medication only	138/565 (24.4%)	1.36 (1.08–1.70)	145/568 (25.5%)	1.27 (1.01–1.59)	57/625(9.1%)	1.59 (1.13–2.25)
Inpatient/outpatient diagnosis	32/150 (21.3%)	0.99 (0.63–1.54)	39/150 (26.0%)	1.25 (0.84–1.86)	15/165 (9.1%)	1.42 (0.70–2.89)

^*^ CI confidence interval; RR, relative risk. Relative risk was estimation by inverse probability of treatment weights (IPTW) weighted Poisson regression model, weighed by propensity score and adjusted for hospital volume (low, median, or high), preoperative chemotherapy (yes/no), preoperative radiotherapy (yes/no), operation type (curative/palliative), history of mental disorder (yes/no).

Perioperative psychiatric morbidity was statistically significantly associated with approximately 2-fold increased risk of overall and cancer-specific mortality (HR = 1.97, 95% CI 1.72–2.26 for overall and HR = 1.94, 95% CI 1.67–2.24 for cancer-specific mortality) (Table 4). Preoperative psychiatric morbidity alone had little impact on the long-term mortality, whereas pre- and post-operative psychiatric morbidity as well as postoperative psychiatric morbidity were both associated with elevated risk of long-term morality. Similar HRs were obtained when patients with and without psychiatric disorder history were analyzed separately (Table 4). Analyses restricted to hospital diagnosed psychiatric morbidity led to similar results (HR = 1.42; 95% CI [1.03–1.94] for overall mortality and HR = 1.54; 95% CI [1.09–2.17] for cancer-specific mortality).

**Table 4:** Hazard ratios and 95 % confidence intervals (CIs) for cancer-specific death occurred after 90 days of surgery among patients with different psychiatric statuses, subgrouped by the history of psychiatric disorder

Psychiatric morbidity	Hazard ratios (95% CI)*		
	All patients alive at least for 90 days after surgery (*n* = 2,485)	Patients with history of psychiatric disorder (*n* = 490)	Patients without history of psychiatric disorder (*n* = 1,995)
**Outcome: overall mortality**			
Overall psychiatric morbidity			
No	Reference	Reference	Reference
Yes	1.97 (1.72–2.26)	1.51 (1.03–2.22)	2.08 (1.80–2.41)
Specified by time of occurrence			
No	Reference	Reference	Reference
Preoperative psychiatric morbidity	0.89 (0.67–1.19)	0.99 (0.56–1.78)	0.86 (0.60–1.21)
Pre- and post- operative (continued) psychiatric morbidity	1.52 (1.24–1.86)	1.46 (0.95–2.22)	1.57 (1.24–2.00)
Postoperative psychiatric morbidity only	3.22 (2.68–3.85)	2.92 (1.63–5.23)	3.30 (2.72–3.99)
**Outcome: cancer-specific mortality**			
Overall psychiatric morbidity			
No	Reference	Reference	Reference
Yes	1.94 (1.67–2.24)	1.31 (0.86–1.97)	2.08 (1.78–2.42)
Specified by time of occurrence			
No	Reference	Reference	Reference
Preoperative psychiatric morbidity	0.83 (0.53–1.30)	0.68 (0.38–1.31)	0.90 (0.65–1.17)
Pre- and post- operative (continued) psychiatric morbidity	1.43 (1.15–1.77)	1.31 (0.84–2.06)	1.46 (1.13–1.89)
Postoperative psychiatric morbidity only	3.32 (2.75–4.01)	2.86 (1.52–5.36)	3.42 (2.80–4.18)

^*^ Evaluated by inverse probability of treatment weights (IPTW) weighted Cox proportional hazards regression model where psychiatric morbidity (or specific type of psychiatric morbidity) was included as a time-varying variable, weighed by propensity score and adjusted for hospital volume (low, median, or high), preoperative chemotherapy (yes/no), preoperative radiotherapy (yes/no), operation type (curative/palliative), history of mental disorder (yes/no), postoperative general complication (yes/no), and postoperative surgical complication (yes/no).

Separate analyses for gastric cancer patients and esophageal cancer patients got very similar results as the main analysis (data not shown). Sensitivity analyses that excluded psychiatric events occurred during 30 or 60 days before death or among patients treated with curative operations didn't change the findings described above. Likewise, the application of alternative propensity score-adjusted regression models also resulted in similar estimates (data not shown).

## DISCUSSION

The impact of psychiatric morbidity on the prognosis of esophageal and gastric cancer patients after surgery was studied in this unique and complete nationwide cohort, based on prospectively collected clinical data of high validity. Our results indicate that regardless of previous history of psychiatric disorders, the presence of perioperative psychiatric morbidity, from two years before to two years after surgery, has a deleterious effect on both short-term and long-term outcomes of surgery for esophageal or gastric cancer patients. More specifically, preoperative psychiatric morbidity increased the risk of having postoperative complications within 30 days by 30%, which consequently conferred a 57% elevated risk of 90-day mortality after surgery. Moreover, both pre- and post- operative psychiatric morbidity seemed to contribute to a poorer long-term survival, after taking other important prognostic factors into consideration (including pre-chemo/radiotherapy, postoperative complications); whereas the greatest risk increase for overall and cancer-specific mortality was observed among patients who developed psychiatric morbidity only after their surgical procedures (about 3-fold increase compared to psychiatric morbidity-free group).

To a varying extent, increased long-term mortality has also been consistently reported among cancer patients with psychiatric disorder by the majority of observational investigations [14, 15]. Correspondingly, evidence from clinical randomized trials have also suggested a survival advantage for cancer patients who received effective psychological interventions [10]. Regarding patients opted for surgical therapy, a recent Swedish study [13] reported an adverse effect of psychiatric morbidity on long-term mortality for esophageal cancer patients, based on analyses with limited disease/treatment- related information. Our results, on one hand, further corroborated their conclusion by adding more detailed personal or disease/treatment-related factors into analyses, through the application of propensity score and weighted multivariate regression models. On the other hand, we advanced analyses further by involving various short-term surgical outcomes, and extended the scope to include patients with gastric cancer, as well as the ones with preexisting psychiatric disease (20% of all operated patients). Hereby, we were able to reach more robust and comprehensive picture of how the perioperative psychiatric morbidity can affect oncological outcomes among surgically treated patients with these aggressive malignancies. Particularly, in line with prior studies [16], we identified psychiatric morbidity that emerged *de novo* after surgery as the most deleterious type of psychiatric morbidity, with regard to long-term mortality, followed by the continued psychiatric morbidity that appeared both before and after surgery. The underlying mechanism for this phenomenon is still unclear. The adverse effects of postoperative psychiatric morbidity on the compliance and tolerance to oncological treatments in conjunction with, or subsequent to surgery can be one possible explanation. Anyway, given that a large proportion of cancer patients [13, 16] (approximately 40% of operated gastric/esophageal cancer patients based on our data) experienced postoperative psychiatric morbidity that might undermine their survival outcomes, further deliberations are much needed to clarify the necessity and cost-effectiveness of an integrated psychiatric care to regular medical management after cancer surgery.

The impact of psychiatric morbidity on short-term prognosis of surgery remains largely unknown. In the present study, we showed that not only was the risk of postoperative complications significantly increased among patients with psychiatric morbidity, but more importantly this was also followed by an increased risk for 90-day mortality (our analyses indicated that the association between preoperative psychiatric morbidity and 90-day death was medicated by the presence of postoperative complications). Therefore, the obvious clinical message here is that an enhanced awareness and availability of early psychiatric supports (ideally from diagnostic workup period) needs be instituted for these cancer patients. One recent study focusing on operated rectal cancer patients, however, failed to demonstrate the association between psychiatric morbidity (defined by inpatient diagnosis only) and poorer short-term outcomes (postoperative complication and length of hospital stay) [16]. Due to the lack of other comparable data, explaining this difference should be with caution. It's possible the prevalence and severity of psychiatric morbidity highly depend on the type of cancer studied, indicating differences in etiology, inborn biological aggressiveness, treatment regimens, and prognosis. Also, factors that important for premorbid infirmity may well be related to demographic background, such as alcohol consumption/smoking habit, life styles, and a variety of socioeconomic factors.

The major strength of our study is the large-scale population-based cohort design, with all information regarding psychiatric morbidity and surgical outcomes collected prospectively and independently. The linked data from death and emigration registers ensured the completeness of follow-up information. The availability of detailed questionnaire data from the quality register enabled considerations of a wide range of personal and disease/treatment-related factors during the analysis —this is extremely important since the associations between many of these covariates (e.g. age, marital status, comorbidity, surgical complications) and psychiatric morbidity, especially postoperative psychiatric morbidity, have been acknowledged by previous studies [13, 16, 17].

Notable limitations include the late establishment of Swedish Outpatient Register (2001-) and Drug Register (2005-), which may cause underestimated proportion of patients with previous psychiatric disorders. However, since we observed similar results among cancer patients with and without preexisting psychiatric diseases, it's very unlikely that such effect could bias our results. Further, the use of psychiatric medication for psychiatric morbidity identification increased the possibility of capturing undiagnosed patients who virtually experienced difficulties in emotional responses [18], at the cost of having possible misclassification induced by this broad definition (considering that antidepressant drug may not be exclusively used for psychiatric problems among cancer patients). But such concern was partly relieved since similar results were yielded from our subanalyses where psychiatric morbidities identified by inpatient/outpatient diagnosis and prescribed psychiatric drug were examined separately. Moreover, although we have attempted to reduce the possibility of reverse causality (psychiatric morbidity caused by facing death-the outcome, instead of by cancer diagnosis/treatment) by removing all psychiatric events within 30/60 days of death, there is no way to evaluate whether the change of cancer disease itself (i.e., recurrence) has contributed to the observed association or not. Further studies with such available data on tumor re-assessment are highly warranted.

In conclusion, our results indicate that irrespective of previous history of psychiatric disorders, the presence of psychiatric dysfunction, occurring within two years either before or after gastro-esophageal cancer surgery, could worsen both short-term and long-term surgery outcomes among patients with gastric or esophageal cancers. Future investigations on the cost-effectiveness of perioperative psychiatric supports among these patients are urgently needed.

## MATERIALS AND METHODS

### Database and study design

The present study was based on the National Registry for Esophageal and Gastric cancer (NREV), which includes all patients with a diagnosis of esophageal or gastric cancer in Sweden. Details about this register have been described elsewhere [2]. In brief, the register was officially launched in 2006, and recruited patients from all centers diagnosing gastric and esophageal cancers in Sweden. The register was cross-checked annually against the Swedish Cancer Register, which has almost 100% completeness of all newly diagnosed cancers in Sweden since 1958, to identify potentially missed patients. In case of lacking coherence, a reminder was then sent to the responsible physicians or clinics in order to complete the registration of such specific patient. According to a recent report, the NREV database, on average, reached a coverage rate of 92% [2].

Comprehensive information regarding the diagnostic work-up, surgical treatment (including operation type, intraoperative blood loss, operation time, etc.), and postoperative follow-ups (e.g. complication, length of hospital stay) was prospectively collected through questionnaires (filled in online by the responsible physician). The register was further cross-linked to the nationwide Cause of Death, Patient, Prescribed Drug and Migration Registers, which provided information on follow-up outcomes of these patients.

In total, 2,747 patients with esophageal or gastric cancer that were operated during 2006–2012 were included. We excluded patients with conflicting information (dead or emigrated before diagnosis, *n* = 2), leaving 2,745 patients in the present analyses. All patients were followed until death, emigration out of Sweden, or December 31, 2012, whichever occurred first. This study was approved by the Regional Ethical Review Board in Stockholm, Sweden (Dnr 2013/596-31/3).

### Identification of psychiatric morbidity

Since we observed clearly elevated need of psychiatric care (assessed by the numbers of hospital visits concerning a psychiatric disorder or prescriptions of psychiatric medications per person-year) among all NREV patients from two years before to two years after surgery (Supplementary Figure 1), in the present study, we defined perioperative psychiatric morbidity as having a clinical diagnosis of psychiatric disorder from two years before to two years after surgery, regardless of a history of earlier psychiatric disorder. We used both the Swedish Patient Register and the Prescribed Drug Register to identify psychiatric morbidity—any inpatient or outpatient hospital visit with a psychiatric disorder diagnosis was counted, using the 7–10th Swedish revisions of ICD codes (ICD7-9: 290-319; ICD-10: F10-F99). To complement the diagnosis from hospital-based specialist care alone, we additionally assessed the use of psychiatric medications, through linking to the Prescribed Drug Register, which documents all drugs dispensed in all pharmacies in Sweden since July 1, 2005 [19]. The related psychiatric medications included antidepressants (ATC code: N06A), antipsychotics (N05A), and anxiolytics (N05B). Perioperative psychiatric morbidity was later categorized as preoperative (if occurred during the two years before the operation) or postoperative (if occurred during the two years after the operation). We also separately analyzed the psychiatric morbidity identified by hospital visit or prescribed medication. To assess the potential impact of previous psychiatric disorders on the studied associations, we also identified the history of psychiatric disorders, i.e. psychiatric diagnosis or use of psychiatric medications more than two years before operation, for subgroup analyses.

### Outcome ascertainment

Short-term outcomes of interest, including general complications within 30 days (severe pneumonia, sepsis, severe cardiovascular complications, pulmonary embolism, and other sever general complications), surgical complications within 30 days (hemorrhage, anastomotic leak, abscess, thoracic duct injury, nerve palsy, and other severe surgical complications), hospital stay (< 14 days/≥ 14 days), and 30/60/90-day mortality were retrieved from NREV database. Information on long-term outcomes, including overall and cancer-specific mortality beyond 90 days after surgery, was obtained from Cause of Death Register with information on date as well as causes of death [20].

### Statistical analysis

To account for potential confounders in the analyses, we first calculated the propensity score by estimating the probability of perioperative psychiatric comorbidity (the exposure) conditioning on a set of patient characteristics for each patient, using a logistic regression model. Variables used in the models included age, sex, marital status (single, married, divorce, widow/widower), education level (< 9 years, 9–12 years, or > 12 years), physical status (The American Society of Anesthesiologists (ASA) classification < 2, 2 and above), cancer type (esophageal/gastric cancer), cancer stage (0–II stage, III stage, or IV stage), and Charlson comorbidity index [21] (0–2, or ≥ 3, calculated according to relevant diseases occurred before the date of the operation, excluding the gastric/esophageal cancer diagnosis itself). This score was subsequently incorporated into regression models in order to balance the distribution of these covariates between exposed and unexposed patients [22, 23]. We ascertained the appropriateness of the generated propensity score through checking the overlap in score distribution between exposed and unexposed groups (common support) [24, 25]. After applying the inverse probability of treatment weights (IPTW) to the all patients (each patient was assigned with a weight presenting the stabilized inverse propensity score), we performed balance assessment using standardized bias [22].

The relative risks (RRs) and 95% confidence intervals (CIs) of short-term outcomes were evaluated by the IPTW-weighted Poisson regression model. The exposure of this analysis was preoperative psychiatric morbidity (i.e., during the two years before surgery), and was further categorized by identification approach as ‘inpatient/outpatient hospital diagnosis’ or ‘psychiatric medication alone’. We adjusted for history of psychiatric disorders more than two years before operation (yes/no), hospital volume (low, median, or high), operation type (curative/ palliative), preoperative chemotherapy (yes/no), preoperative radiotherapy (yes/no), and operation time (hours). In particular, for the analysis of 90-day mortality after surgery, we further adjusted for general and surgical complications within 30 days (yes/no) to assess the potential pathways between preoperative psychiatric morbidity and 90-day mortality.

For patients alive 90 days after operation (*n* = 2,485), we used an IPTW-weighted Cox proportional hazards model [26, 27] to estimate the hazard ratios (HRs) for overall and cancer-specific death in relation to perioperative psychiatric morbidity, after controlling for all covariates stated above. Prior investigations have demonstrated that this model could minimize survival bias in time-to-event analyses [28]. The underlying timescale is time since 90 days after operation, and the exposure was included as a time-varying variable—i.e. patients with psychiatric morbidity from two years before to 90 days after operation were classified as exposed from the start of follow-up, whereas patients with psychiatric morbidity between 90 days and two years after operation were classified as unexposed before psychiatric morbidity and exposed after psychiatric morbidity; the other patients were always classified as unexposed. In the subanalyses where the type of psychiatric morbidity was further classified as ‘preoperative’, ‘both pre- and post- operative’, and ‘postoperative’ psychiatric morbidity, the change from ‘pre’ to ‘pre and post’ group was also allowed. We further repeated the analyses by restricting to perioperative psychiatric morbidity captured by ‘inpatient/outpatient hospital diagnoses’. Additionally, we conducted a sensitivity analysis by deleting the psychiatric events that occurred within 30 or 60 days of death, in order to test if the results could be severely biased by the psychiatric problems emerging as a consequence of the fear of approaching death (i.e., reverse causality).

We assessed the potential modification effect of history of psychiatric morbidity more than two years before operation on the studied associations, by estimating patients with or without such history separately. Also, all abovementioned analyses were repeated among subpopulations, where gastric cancer patients and esophageal cancer patients were separated. We additionally performed a sensitivity analysis by restricting the analyses to patients that received an operation with curative intent (*n* = 2,222). Lastly, we applied alternative propensity score-adjusted, instead of IPTW-weighted, regression models, to verify the results obtained from main analyses.

A *p* value less than 0.05 was considered to be statistically significant. All analyses were conducted in SAS statistical software, version 9.4 (Cary, NC).

## SUPPLEMENTARY MATERIALS FIGURE



## Abbreviations

ASA: The American Society of Anesthesiologists
CI: confidence interval
HR: hazard ratio
ICD: International Classification of Diseases codes
IPTW: Inverse probability of treatment weights
NREV: National Quality Register for Esophageal and Gastric Cancers
