# Supplementary material for: Psychiatric morbidity and its impact on surgical outcomes for esophageal and gastric cancer patients: A nationwide cohort study
Source: Oncotarget. 2017 Jun 2;8(46):81305–14. doi: 10.18632/oncotarget.18347 (PMC5655284; doi:10.18632/oncotarget.18347)
Supplement: Supplementary file 1 [file oncotarget-08-81305-s001.pdf]

Psychiatric morbidity and its impact on surgical outcomes for esophageal and gastric cancer patients: A nationwide cohort study

Supplementary Materials

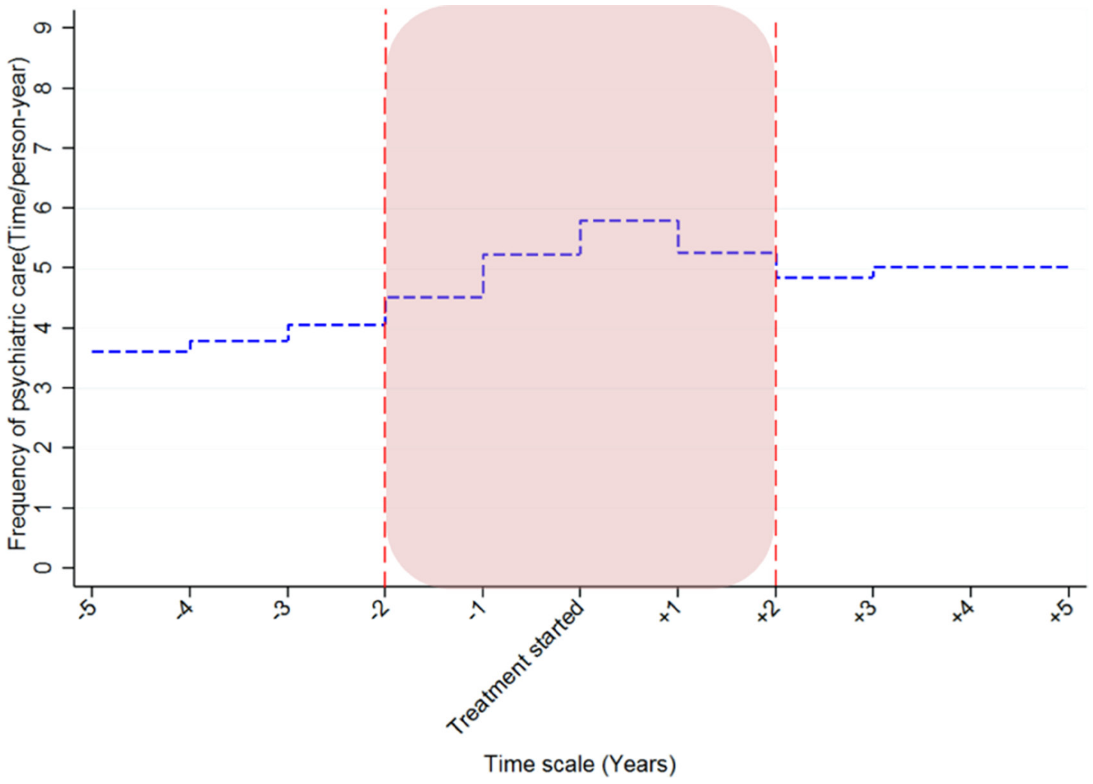

Supplementary Figure 1: Changes of the frequencies of psychiatric care* during cancer treatment time frame (± 5 years) among patients with gastric and esophageal cancer (*n* = 7,080). *assessed by the numbers of hospital visits concerning a psychiatric disorder or prescriptions of psychiatric medications per person-year.
